# A multifaceted quality improvement intervention for CVD risk management in Australian primary healthcare: a protocol for a process evaluation

**DOI:** 10.1186/s13012-014-0187-8

**Published:** 2014-12-17

**Authors:** Bindu Patel, Anushka Patel, Stephen Jan, Tim Usherwood, Mark Harris, Katie Panaretto, Nicholas Zwar, Julie Redfern, Jesse Jansen, Jenny Doust, David Peiris

**Affiliations:** The George Institute for Global Health, University of Sydney, Sydney, NSW 2006 Australia; University of Sydney, Sydney, NSW 2006 Australia; University of New South Wales, Sydney, NSW 2052 Australia; Queensland Aboriginal and Islander Health Council, 21 Buchanan St., West End, QLD 4101 Australia; Bond University, 14 University Dr, Robina, QLD 4226 Australia

**Keywords:** Knowledge translation, Process evaluation, Quality improvement intervention, Clinical decision support systems, Cardiovascular prevention, Cardiovascular risk, Primary healthcare, Mixed methods, Theory-based

## Abstract

**Background:**

Cardiovascular disease (CVD) is the leading cause of death and disability worldwide. Despite the widespread availability of evidence-based clinical guidelines and validated risk predication equations for prevention and management of CVD, their translation into routine practice is limited. We developed a multifaceted quality improvement intervention for CVD risk management which incorporates electronic decision support, patient risk communication tools, computerised audit and feedback tools, and monthly, peer-ranked performance feedback via a web portal. The intervention was implemented in a cluster randomised controlled trial in 60 primary healthcare services in Australia. Overall, there were improvements in risk factor recording and in prescribing of recommended treatments among under-treated individuals, but it is unclear how this intervention was used in practice and what factors promoted or hindered its use. This information is necessary to optimise intervention impact and maximally implement it in a post-trial context. In this study protocol, we outline our methods to conduct a theory-based, process evaluation of the intervention. Our aims are to understand how, why, and for whom the intervention produced the observed outcomes and to develop effective strategies for translation and dissemination.

**Methods/Design:**

We will conduct four discrete but inter-related studies taking a mixed methods approach. Our quantitative studies will examine (1) the longer term effectiveness of the intervention post-trial, (2) patient and health service level correlates with trial outcomes, and (3) the health economic impact of implementing the intervention at scale. The qualitative studies will (1) identify healthcare provider perspectives on implementation barriers and enablers and (2) use video ethnography and patient semi-structured interviews to understand how cardiovascular risk is communicated in the doctor/patient interaction both with and without the use of intervention. We will also assess the costs of implementing the intervention in Australian primary healthcare settings which will inform scale-up considerations.

**Discussion:**

This mixed methods evaluation will provide a detailed understanding of the process of implementing a quality improvement intervention and identify the factors that might influence scalability and sustainability.

**Trials registration:**

12611000478910.

## Background

### Cardiovascular disease burden

Non-communicable diseases (NCDs)—including cardiovascular diseases (CVD), cancers, respiratory diseases, and diabetes mellitus—are the leading cause of death worldwide [1,2]. It is predicted that by 2030, NCDs will account for 75% of all deaths with the largest proportion of deaths attributed to CVD [3]. Globally, approximately 17 million (30%) deaths per year and 151 million disability-adjusted life years (DALYs) are caused by CVD [4].

In Australia, CVD is responsible for 34% of deaths and 18% of the burden of disease and injury, making it the largest contributor to health system expenditure [5]. Aboriginal and Torres Strait Islander people experience a greater CVD burden than other Australians, and this is a major contributing factor to the 10-year gap in life expectancy [6,7]. The majority of CVD is caused by modifiable risk factors, which include blood pressure, lipids, diabetes, body mass index (BMI), tobacco smoking, alcohol use, unhealthy diet, physical inactivity, and psychosocial stress [8]. Recent modelling suggests that a 25% reduction in the prevalence of six NCD risk factors alone (tobacco, alcohol, salt, blood pressure, obesity, and glucose) could reduce global disease burden by 25% in the next 10 years [9].

### Evidence practice gaps

International clinical guidelines recommend that assessment for CVD prevention and management should be based on a combination of risk factors (the ‘absolute risk’ approach) rather than treating risk factors such as elevated blood pressure and cholesterol in isolation [10-13]. Absolute risk calculation estimates an individual’s risk of a CVD event over time based on modifiable and non-modifiable risk factors such as age and gender. This enables early identification, management, and primary prevention of CVD for individuals at high risk. In combination with well-established secondary prevention recommendations for people who have experienced a previous CVD event, the absolute risk approach offers considerable potential for reducing CVD burden [14].

Despite the widespread availability and consistency of these guidelines and the availability of validated risk prediction equations, there are large evidence practice gaps. Health professionals tend to use these guidelines and equations sporadically and inconsistently [15,16]. In the Australian context, studies have found that only 50% of adults attending primary healthcare have been screened for CVD risk in accordance with guideline recommendations, and only 40% of those identified as high risk have been prescribed recommended medications [17,18]. International studies have similarly demonstrated that a minority of people are being provided with appropriate screening measures and preventive treatments [19]. The majority of CVD events can potentially be averted with adequate implementation of established treatments and interventions that are effective, efficient, and universally accessible in Australian primary healthcare settings. Primary healthcare is the ‘front-line’ for effective prevention and management of the increasing burden of chronic diseases. With 88% of Australians visiting a general practitioner each year, the opportunities to improve primary and secondary prevention of CVD at this level are great [20].

### Knowledge translation strategies

Given the magnitude of these evidence practice gaps in CVD prevention, effective quality improvement (QI) innovations that support health services to improve their outcomes are urgently needed. QI is a multidimensional concept that focuses on improving the efficiency and process of a program, service, or organisation, resulting in improved health outcomes [21,22]. The Agency for Healthcare Research and Quality (AHRQ) has identified nine broad QI strategies to improve quality of care: *provider reminder systems, facilitated relay of clinical data to providers, audit and feedback, provider education, patient education, promotion of self-management, patient reminder systems, organisational change, and financial incentives, regulation, and policy* [23]. An increasingly important component of QI strategies is an adoption of health information technologies (HITs). Meaningful use of electronic health records (EHRs), computerised provider order entry systems, and electronic decision support (EDS) are increasingly recognised as key enablers to improvements in quality and delivery of healthcare [24]. However, the overall impact of computerised QI interventions to improve CVD burden has been limited, and effects on patient outcomes remain unclear [25-27].

Whilst QI strategies have been well characterised, there is relatively little knowledge translation research to help guide how these strategies can be optimally implemented into routine practice [28,29]. The Canadian Institute of Health Research defines knowledge translation as ‘*the exchange, synthesis and ethically sound application of knowledge—within a complex system of interactions among researchers and users—to accelerate the capture of the benefits of research for patients through improved health, more effective services and products and a strengthened health care system*’ [30]. Whilst it is critical that QI interventions are robustly assessed for effectiveness, equally important is a detailed understanding of how those QI interventions are implemented, using process and economic evaluations. This will allow a deeper understanding of how the intervention worked/did not work, in which contexts was it most effective/ineffective, and why. Given QI interventions are inevitably complex in nature, robust evaluation requires the use of multiple theories and frameworks to answer these questions. By using knowledge translation frameworks, we are able to better identify active ingredients of the intervention that change behaviour, causal mechanisms of change, effective modes of delivery, and the intended population or target [31]. This will enable promotion and integration of the interventions into clinical practice, health systems, and policy.

### The treatment of cardiovascular risk in primary care using electronic decision support (TORPEDO) study

**Figure 1:**
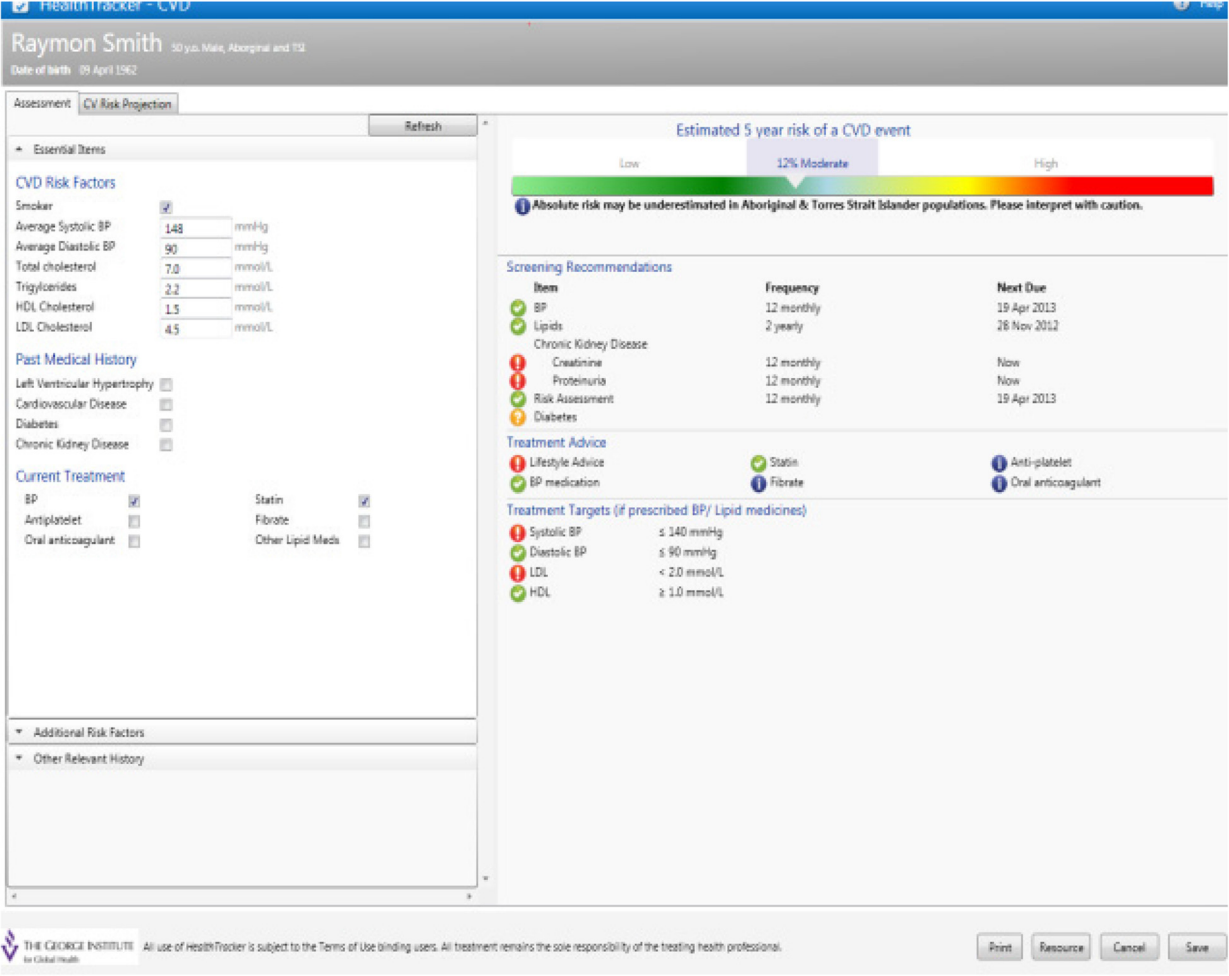
**Real-time decision support interface.**

**Figure 2:**
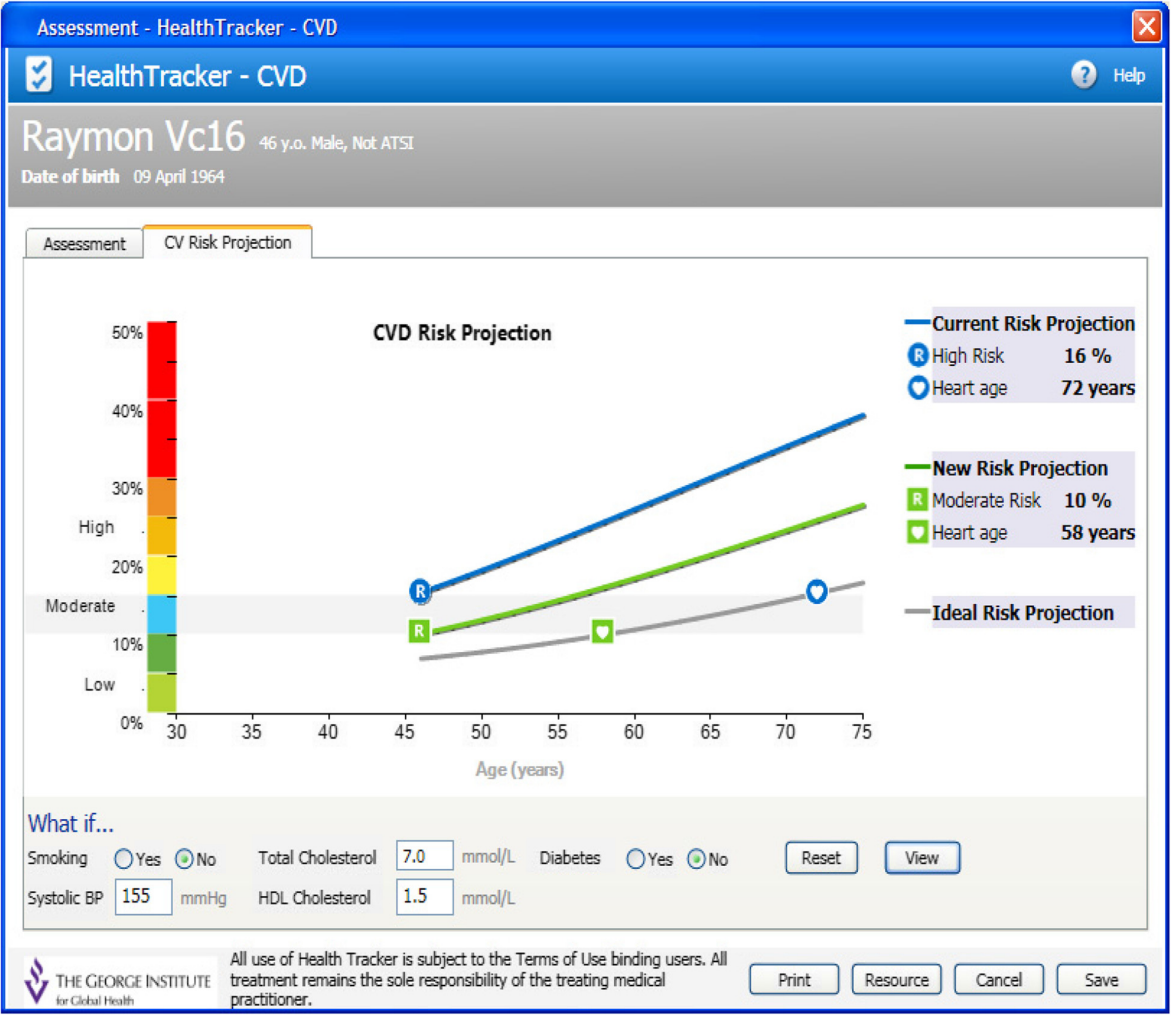
**Patient-oriented risk communication interface.**

**Figure 3:**
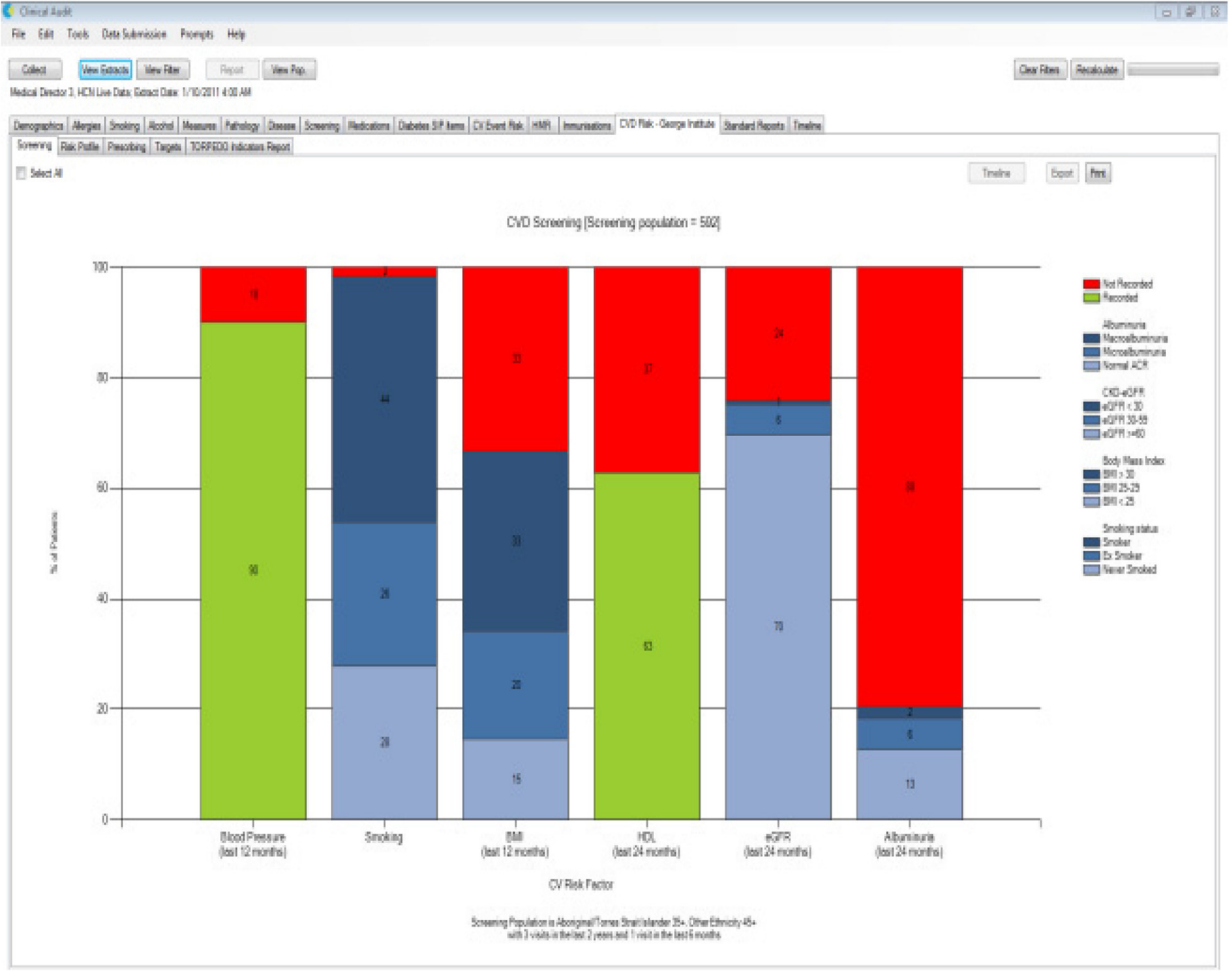
**Automated data extraction tool—sample health service performance on CVD risk factor screening.**

**Figure 4:**
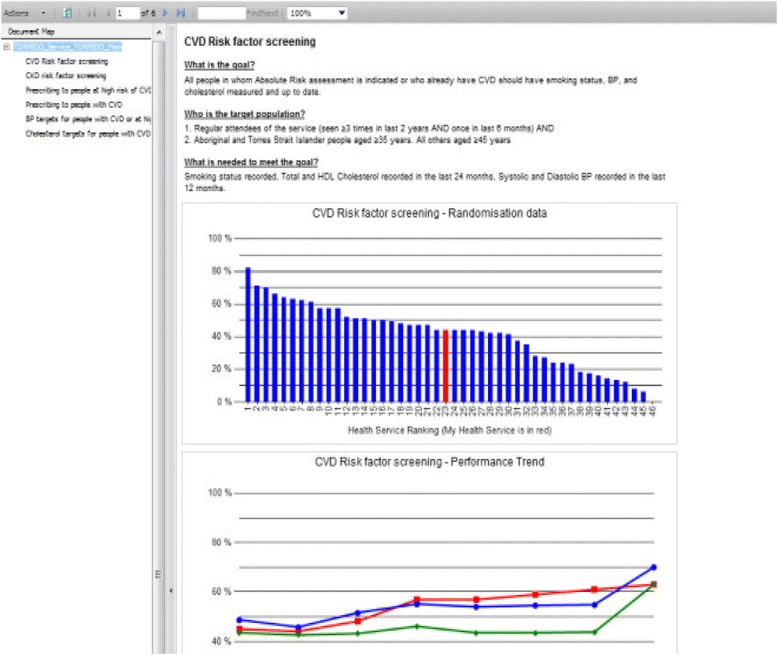
**Quality improvement portal.**

There have been few randomised evaluations of QI interventions in the Australian primary healthcare setting. We designed a multifaceted QI intervention for CVD risk management in Australian primary healthcare. The intervention drew on two established QI mechanisms: 1) electronic decision support and 2) audit and feedback (summary of the clinical performance over a specified period of time) [32-37]. The intervention was evaluated in the TORPEDO study, a cluster-randomised controlled trial (cRCT) involving 60 health services. Details of the trial are published elsewhere [38]. In brief, the system was integrated with the healthcare provider’s EHRs, and included (1) a real-time decision support interface using an algorithm derived from several evidence based national guidelines (Figure 1); (2) a patient risk communication interface which included ‘what if scenarios’ to show the benefits from particular health risk factor improvement during a consultation (Figure 2); (3) an automated clinical audit tool for extraction of data and review of health service performance (Figure 3); and (4) a web portal where services can view peer-ranked performance over time (Figure 4). Healthcare providers could use the point-of-care tool as part of a routine clinical consultation. For the audit and feedback component, quality indicators were developed for patients who had visited the health service at least three times in the preceding 2 years and once in the preceding 6 months. The population studied was based on national guideline recommendations for CVD risk screening and included Aboriginal and Torres Strait Islander people over 35 years and all others over 45 years [38].

Data were collected for 38,725 people from the 60 health services. Overall, when compared with control, the intervention was associated with a 25% relative (10% absolute) improvement in CVD risk factor screening. Overall, there was no significant difference in the prescribing rates of recommended medicines to people at high CVD risk. The intervention was, however, strongly associated with improvements in the sub-group at high CVD risk that was not prescribed with recommended medicines at baseline. There were also improvements in intensification of existing, recommended medication regimens. There were modest improvements in attaining blood pressure targets but no differences in other clinical outcomes. The improvements in recommended prescriptions to high risk patients were not accompanied by increased prescribing rates for patients at low risk [39].

Although the intervention exhibited significant improvements in some outcomes, it remains unclear how this intervention was actually used in practice and what factors promoted and hindered its use. Answers to these questions are critical in order to inform future directions for its implementation and for implementation of similar interventions in other settings.

In this paper, we outline our protocol for a theory-based process evaluation of the TORPEDO intervention. The broad objectives are to understand how, why, and for whom the intervention produced the observed outcomes and to develop effective strategies for translation and dissemination. The evaluation will identify which intervention components promoted or had minimal impact at the provider, patient, and system levels and the mechanism of change. It will also identify the contextual influences on the delivery of the intervention and its outcomes. The specific objectives are the following:

1. To understand whether intervention effects are sustained in a post-trial setting;
2. To identify implementation barriers and enablers;
3. To understand how CVD risk is communicated in the doctor/patient interaction both with and without the use of the intervention; and
4. To identify cost considerations for delivering the intervention at scale in the Australian primary healthcare system.

## Design and methods

Taking a mixed methods approach, we will conduct four discrete but inter-related studies to address our study objectives. Specifically, we will adopt an explanatory sequential design whereby the qualitative data analysis will be used to gain a better understanding of the quantitative findings [40].

### Logic model

**Figure 5:**
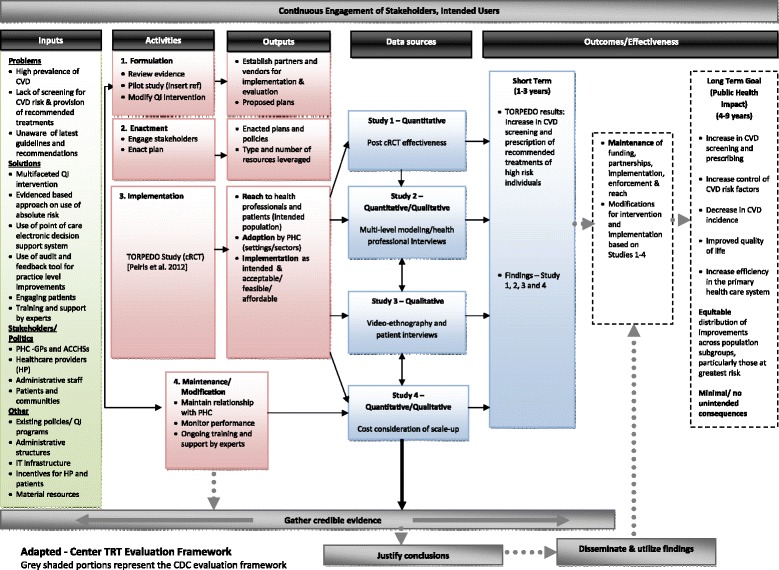
**Logic model for TORPEDO process evaluation.**

Drawing on the RE-AIM framework, a logic model was developed to assist in the planning, conduct, and evaluation of the research components (Figure 5) [41-44]. The model assesses five dimensions of the intervention at different levels (individual, health service/clinic or organisation, and community/population): (1) participant *R*each; (2) *E*ffectiveness of the intervention; (3) *A*doption by the target health service; (4) *I*mplementation fidelity, costs, and adaptations made during delivery; and (5) *M*aintenance of intervention effects over time. The model identifies and describes inputs, activities, outputs, and outcomes of the intervention [45]. The four objectives have been mapped onto the relevant components of the RE-AIM framework.

The logic model will be underpinned by three theoretical perspectives—realist evaluation [46], the theoretical domain framework (TDF) [47,48], and normalisation process theory (NPT) [49,50].

### Realist evaluation

Realist evaluation seeks to answer the question *what works*, *for whom*, *and in what circumstances?* [46]. This is accomplished by identifying and understanding the underlying mechanisms by which the intervention succeeds or fails in varying contexts to produce the patterns of outcomes. Therefore, unpacking of the underlying generative mechanisms of the intervention and its effects is contingent on understanding the features of the context (i.e. roles and relationships of personnel at health services, IT infrastructure, economic conditions, demographic, motivation and skills of health professionals, etc.) [46,51,52].

### Theoretical domain framework

Successful implementation of evidence-based guidelines and QI interventions depends largely on changing the behaviour of healthcare professionals and patients, who are influenced by external (i.e. organisational, environmental, resources) and internal (i.e. motivation, capability) factors. TDF is a consensus of numerous behaviour change models and comprises 14 domains derived from psychological and organisational theory (knowledge, skills, social/professional role and identity, beliefs about capabilities, optimism, beliefs about consequences, reinforcement, intentions, goals, memory attention and decision process, environmental context and resources, social influences, emotion, and behavioural regulation) [47,48,53].

### Normalisation process theory

In order for healthcare innovation and technology to become routinely embedded in every day work, we need to understand how the innovation is integrated within the existing practices of a healthcare organisation. NPT assists in understanding how health professionals implement an intervention. NPT identified four main components: (1) coherence (sense making by participants), (2) cognitive participation (commitment and engagement by participants), (3) collective action (the work participants do to make the intervention function), and (4) reflexive monitoring (the degree to which participants reflect on or appraise the intervention [49,54,55].

### Study 1: Post-trial effectiveness of the intervention (objective 1)

#### Aim

To assess the effects of the intervention at one year following completion of the cRCT.

#### Methods

At the end of study follow-up visit for the TORPEDO study, all 60 health services have the option to either continue the use of the intervention or have the intervention implemented for use in the usual care health services for an additional 12 months. A post-trial clinical audit data extraction at minimum of 24 months from baseline will be conducted for the health services expressing interest to use the intervention. The objective of this study is to assess the impact of the use of the intervention over time on the two primary outcomes: (1) proportion of CVD risk factor screening and (2) proportion of appropriate medication prescription in the high risk individuals for the intervention arm and usual care arm at post-end of study following baseline.

#### Analysis

Log-binomial regression will allow direct estimation of risks and risk ratios (i.e. relative risks) on each outcome. The model will be adjusted with intervention (yes vs. no) and time (baseline, 12 and 24 month) as categorical data where baseline is set as reference and interaction between intervention and time. The model will be adjusted with the intervention (yes or no) and with a random centre effect. Chi-square test and 95% confidence intervals (CI) will be computed.

### Study 2: Multilevel modelling study (objective 2)

#### Aim

To determine what patient and health service level variables correlate with the trial outcomes.

#### Methods

Patient variables (age, gender, ethnicity, history of CVD, and diabetes) will be obtained from the automated clinical audit tool, and health service level variables will be collected through Team Climate Inventory (TCI) and Warr-Cook-Wall job satisfaction surveys, customised for use with general practices (GPs) and Aboriginal Community Controlled Health Services (ACCHSs), administered to all general practitioners and other practice and health service staff at the participating 60 sites [56,57]. In addition, health service characteristics such as service size, participation in quality improvement programs, and type of primary healthcare (GP verses ACCHS) will be collected at randomisation.

#### Analysis

TCI is a 44-item questionnaire, and items are rated on a 5-point scale [56]. Job satisfaction is a 15-item questionnaire, and items are rated on a 7-point scale [57]. Multilevel regression model analysis will be conducted to evaluate the influence of team climate and job satisfaction on the primary study outcomes, controlling for patient’s age, gender, practice size, type of PHC, and participation in quality improvement programs. The results will be interpreted within the context of the three conceptual perspectives to better understand what health service factors (if any) are important drivers of use of the intervention in routine practice.

### Study 3: Interview study and video ethnography (objectives 2 and 3)

#### Aim

To identify and understand which intervention components promoted or had minimal impact on behaviour change at the provider and patient levels, the mechanism of change, and contextual influences.

#### Methods

Case study methods will be used to explore system changes over time, through in-depth qualitative data collection involving multiple sources of information. The cases will be individual GPs and ACCHSs participating in the TORPEDO study. Quantitative data obtained from the primary study and the studies 1 and 2 above will be drawn on as part of the analysis of the cases, and new qualitative data will be obtained using semi-structured interviews, video ethnography, and surveys. This will ensure that both intervention effects and implementation processes are comprehensively assessed. It will identify contextual influences, and by drawing on multiple empirical data sources will increase the robustness of the findings [58,59].

We will purposively sample health services to achieve maximum variation in trial primary outcomes, numbers of staff at each site, and type of service (GP versus ACCHS, urban versus rural). We will select six cases from the intervention arm (four GPs and two ACCHSs) and three cases from the usual care arm (two GPs + one ACCHSs). There will be two methods of data collection.

#### Health professional interviews

Semi-structured interviews with site staff will provide us with their knowledge, views, and experience of the implementation of the intervention at their health service. Interview questions have been developed to explore the realist evaluation domains of context, mechanism, and outcome. Some questions include the following: (1) why health staff did/did not use the intervention; (2) how was the intervention used in routine practice and by whom; (3) what were the contextual factors that influenced its uptake; and (4) what impact did it have on the way personnel did their work. We will conduct approximately 20 health professional interviews with general practitioners, nurses, managers, Aboriginal health workers (AHWs), and administrative assistants from within our cases. The final number of interviews will be dependent on thematic saturation [60]. The interviews will take place at the health service, and all interviews will be audio-recorded and transcribed. The interviews will take place face to face with a thematic and topic-centred interview guide. The interview guide will be flexible to allow exploration of emergent themes.

#### Video ethnography

Qualitative data obtained from ethnographic studies can enhance our understanding of how to introduce a technological innovation into healthcare [61,62]. In order to augment our interview data, video ethnography will be used to give us insight into (1) the possible ways general practitioners used the intervention tools; (2) how cardiovascular risk is talked about between general practitioner and patient; (3) how patients receive and interpret this information; and (4) what impact the intervention tools have on the decision-making process, particularly related to recommending and taking medication. Approximately 20% of patients video-recorded (approximately two per general practitioner) will be selected to be interviewed after videotaping of their consultation. Patients agreeing to participate will be interviewed at their home or the health service at a time suitable for the patient.

#### Analysis

Framework analysis will be used to organise the interview data [63]. Key issues and emergent themes will be identified and then a coding framework will be developed to index and chart interview transcripts and videos. Videos will be subject to fine-grain discourse analysis [64]. NVivo 10 software (QSR International) will be used for data management and coding of interview transcripts, field notes, and videos. The analysis will occur simultaneously with data collection. Themes will be developed both deductively (pre-defined themes) and inductively (emerging themes), and then coded in an iterative process to identify patterns, and interpret the meaning of the themes within and across cases. Throughout this process, we will meet regularly with a project working group of expert researchers and collaborators to discuss the theoretical framework within which the data will be collected, coded, and interpreted. Reflexivity will be incorporated into the qualitative analysis process to take into account personal assumptions and biases, so these do not influence the way and the type of data that are collected or the data analysed.

Our three chosen theoretical perspectives will be regularly drawn on to assist with interpretation of the qualitative findings. Using realist evaluation, we expect the analysis to yield insights into particular context-mechanism-outcome configurations that explain patterns associated with use and non-use of the intervention. The TDF will complement these analyses and explore to what extent the intervention influenced behaviour change by various actors (health professionals, managers, and patients). Data will be used to make an assessment of the underlying capacity, motivation, and opportunities of these actors and the extent to which the intervention influenced these areas. NPT will be used to provide a better understanding of the ways in which health services as organisational structures respond to the intervention. Interview codes will be aligned with the four NPT domains of coherence, cognitive participation, collective action, and reflexive monitoring, and it is expected this will facilitate our analyses and derivation of the key messages.

### Study 4: Cost consideration of scale-up (objective 4)

#### Aim

The cost implications for health services to adopt the intervention, and deliver at scale in Australia.

#### Summary

A business model will be developed for health services to adopt and maintain the intervention. We will both quantitatively and qualitatively explore the factors that will influence costs for the various types of health services (i.e. large, medium and small health services, patient load/GP, etc.), capacity constraints within individual practices, the investment needed to adopt the intervention, and the potential returns to the practice in terms of patient care. These will be assessed across a diverse range of practices. This evidence will be obtained through clinical audit data, surveys, and health professional semi-structured interviews. The findings will be used to determine the economic viability of the widespread adoption and implementation of this intervention and inform policy by ascertaining the support that individual practices will need to accomplish these tasks and ultimately the costs to government of scaling up.

## Ethical considerations

The study is approved by The University of Sydney Human Research Ethics Committee (2012/2183) and the Aboriginal Health & Medical Research Council (AH&MRC) of New South Wales (778/11). Participation agreements were signed between the participating health services and the coordinating research institute. De-identified patient level data is being extracted from health service software systems to analyse post-trial outcomes (study 1) and assess health service utilisation costs (study 4). Participants in the TCI and job satisfaction surveys (study 2) and the qualitative study components will be provided with an information sheet and asked to provide written informed consent to participate. Participants will be reassured of the confidential nature of any data collected, and they will be identified by a unique identification number only. Participants will be reminded that they can opt not to answer any questions or can stop interviews or videotaping at any time, and they will have a right to withdraw consent and cease involvement in the study without penalty.

## Trial status

Data collection is underway. Preliminary qualitative data analysis is being conducted contemporaneously with data collection. Quantitative data analysis has not commenced.

## Discussion

Addressing the challenges of CVD burden requires implementation strategies for increasing the uptake of well-established evidence into practice. Our attempt to address this with a multifaceted QI intervention was moderately but not uniformly successful, suggesting the need for a rigorous process evaluation to understand how and in what ways it was taken up in practice. Such evaluations are crucial to understanding how implementation strategies should be applied in non-trial settings.

Multifaceted interventions, by their nature, invariably lead to complex usage patterns which can make interpretation of study outcomes difficult. In order to maximise understanding that is relevant to other settings, process evaluation is therefore critical. The strength of our process evaluation is its multicomponent, multi-theory approach combining diverse study designs to make sense of how this particular knowledge translation strategy was adopted into practice. Further, by examining implementation from multiple perspectives (provider, patient, health services, and system) the findings are expected to provide both micro- and macro-system perspectives which will be of interest to policy makers and implementers. There are two key limitations to our approach (1) the majority of the data collection will occur toward the end of the trial and in the post-trial phase and may miss critical insights gained from early phase adoption processes; and (2) the study setting is limited to Australian primary healthcare settings and therefore may be only of relevance to health systems with similar contexts, financing, workforce structures, and adoption of electronic medical records.

Despite these caveats, the adoption and successful implementation of computerised QI interventions and strategies are the key challenges for healthcare systems worldwide. In 2009, the US government passed the Health Information Technology for Economic and Clinical Health Act as a stimulus to promote and adopt ‘meaningful use’ of information technologies. The Act provided incentive payments to hospitals and individual practices totalling $14–27 billion to adopt EHRs within 3 years to avoid financial penalties. This unprecedented investment is a reflection of the importance of information technology adoption for health systems reform. In Australia, the National E-Health Transition Authority was established in 2010 with a government investment of over $467 million to develop and implement e-health systems nationally. Despite such large publicly funded investments, there remains uncertainty around the factors that will promote successful adoption of computerised QI strategies.

This mixed methods process evaluation, grounded in a theoretical framework, will evaluate the impact of a complex, multifaceted intervention and help us to understand the knowledge translation considerations for use of computerised QI interventions in clinical practice.

## Abbreviations

ACCHS: Aboriginal Community Controlled Health Service
AH&MRC: Aboriginal Health and Medical Research Council
BMI: body mass index
CDS: clinical decision support
cRCT: cluster randomised controlled trial
CVD: cardiovascular disease
EHR: electronic health record
GP: general practice
HIT: health information technology
NCD: non-communicable disease
NHMRC: National Health and Medical Research Council
NPT: normalisation process theory
QI: quality improvement
RE-AIM: reach, effectiveness, adoption, implementation, maintenance
TCI: Team Climate Inventory
TDF: theoretical domain framework
TORPEDO: the treatment of cardiovascular risk in primary care using electronic decision support
